# In Search of Safe Spaces: An Exploratory Study of the Anticipated Help-Seeking Needs and Preferences of Protestant Christian Women in Singapore with Respect to a Hypothetical Abortion Scenario

**DOI:** 10.1007/s10943-023-01766-y

**Published:** 2023-02-22

**Authors:** S. J. Y. Teoh, P. K. Low, J. E. Ramsay

**Affiliations:** School of Social and Health Sciences, James Cook University, Singapore, Singapore

**Keywords:** Protestant, Christian women, Abortion, Stigma, Social support, Help-seeking, Religion

## Abstract

Research suggests that religious beliefs may contribute to abortion stigma, resulting in increased secrecy, reduced social support and help-seeking as well as poor coping and negative emotional consequences such as shame and guilt. This study sought to explore the anticipated help-seeking preferences and difficulties of Protestant Christian women in Singapore with regard to a hypothetical abortion scenario. Semi-structured interviews were conducted with 11 self-identified Christian women recruited through purposive and snowball sampling. The sample was largely Singaporean and all participants were ethnically Chinese females of a similar age range (late twenties to mid-thirties). All willing participants were recruited regardless of denomination. All participants anticipated experiences of felt, enacted and internalized stigma. These were affected by their perceptions of God (e.g., how they see abortion), their personal definitions of “life” and their perceptions of their religio-social environment (e.g., perceived social safety and fears). These concerns contributed to participants choosing both faith-based and secular formal support sources with caveats, despite a primary preference for faith-based informal support and secondary preference for faith-based formal support. All participants anticipated negative post-abortion emotional outcomes, coping difficulties and short-term decision dissatisfaction. However, participants who reported more accepting views of abortion also anticipated an increase in decision satisfaction and well-being in the longer term.

## Introduction

Although the relationship between abortion and mental health outcomes is multifactorial (Bellieni & Buonocore, 2013; Steinberg et al., 2012), research suggests that women denied abortions are at greater risk of mental health issues than those who receive them (Foster et al., 2015). Research also suggests that post-abortion outcomes encompass both long- and short-term psychosocial concerns such as social isolation, feelings of guilt and shame, and symptoms reminiscent of post-traumatic stress disorder (Trybulski, 2006). Painful thoughts and feelings may occur during everyday events or on certain dates and concerns may be ongoing as life events unfold. Thus, social support, a key protective factor in post-abortion psychological recovery (Major et al., 2009), may be necessary on an ongoing, rather than one–off, basis. This study sought to explore how abortion stigma impacts the perceived availability of social support and help-seeking choices of Protestant Christian women in Singapore.

### Abortion and the State

Induced abortion is defined as the medical termination of pregnancy (Norman et al., 2013). Singapore’s Termination of Pregnancy (TOP) Act safeguards female access to an induced abortion^1^ (without the need for partner or parental consent) until the gestational limit of 24 weeks, after which abortion becomes prohibited except in cases of life-threatening harm or grave permanent injury to the woman’s physical or mental health (Ministry of Health (MOH), 2015). Legally, individuals seeking abortion must undergo mandatory counselling, including viewing an abortion procedure video, and a 48-h cooling-off period.

Despite the relative ease of abortion access in Singapore, the abortion rate has been falling since its peak of almost 24,000 cases in 1985 (Tan, 2020) to 4,029 abortions performed for Singaporeans in 2020 (MOH, 2021). This decline has been attributed to various factors including increased birth control usage, decreased stigma of single parenthood, and the inclusion of children born to unwed parents in the Child Development Account, a national savings scheme (Tan, 2020). However, abortion decisions are rarely made based purely on logic. Often, pre-abortion decision-making are influenced by other, more subjective considerations, including the religio-moral narratives one subscribes to, the reactions of other people and difficult emotions upon the discovery of an untimely pregnancy. These, in turn, are influenced by the pervading social discourse surrounding abortion.

### Abortion and Protestant Christianity in Singapore

Viewpoints in the abortion debate range across a spectrum with strongly pro-life and pro-choice views representing the opposing poles. Despite the polarized nature of public discourse on the topic, individuals’ viewpoints may be more nuanced, falling in the grey area in between (e.g., accepting elective induced abortions in some scenarios but not others). Such views may contribute to social and religious narratives of how Christian women are expected to feel, think and choose with regard to abortion and pregnancy.

In the Census of Population 2020 by the Singapore Department of Statistics (2021), Christians comprised 18.9% of the population (aged 15 years and up), making Christianity Singapore’s second largest religious group. Although a politically secular nation, Singaporeans are generally quite religious (Mathews et al., 2019) and religious views exert a significant influence in both private spheres and public discourse, with several Church groups speaking out on moral issues (e.g., Alliance of Pentecostal & Charismatic Churches of Singapore, n.d.).

A summary of key findings in the World Values Survey (Mathews et al, 2021) showed that 66.5% of Protestant Christians believed abortion to be never or seldom justifiable, a proportion higher than the national average and that of non-religious individuals. This suggests that Protestant Christian communities are likelier to hold disapproving views of abortion, as reflected in a 2010 statement by the National Council of Churches in Singapore which stated that “the embryo or fetus is a human being, made in the image of God… (and that it) cannot countenance the destruction of a foetus even in the context of legalised elective abortion”.

On a more macro-level, the effects of religion (including Christianity) on any national discourse may be strengthened by the “high regard and strong levels of confidence in (religious institutions), on par with the elected members of Parliament of Singapore” in which the “vast majority of Singaporeans” hold them (Mathews et al., 2019, p. 76). Mathews et al. (2019) also noted that it is “imperative to understand the relationship between religion and attitudes towards secularism in the context of Singapore” (p. 141), citing the extent of polarization between religious and non-religious groups abroad (e.g., regarding abortion’s legality).

On a more micro-level, the effects of religio-moral narratives on an individual’s attitude towards abortion may be amplified by the social nature of Christianity in Singapore. Christians here are likelier than other religions to participate in organized worship (Mathews et al., 2019). As of 2014, 54% of women in the USA who had abortions identified as Christian (30% Protestant, 24% Catholic) (Jones, 2020). There are no similar Singapore-based statistics; however, this does not preclude Protestant Christian women in Singapore from abortion. It is important then to understand how their daily lived experiences are impacted by both the abortion-related religious narratives and the highly social nature of their religious social groups.

This impact is examined in terms of Herek et al.’s (2009) social psychological framework of stigma which comprises internalized, felt and enacted stigma. Internalized self-stigma may make it more psychologically difficult for women to claim their religious affiliation or to draw emotional comfort from it (Frohwirth et al., 2018). Felt and enacted stigma may compromise an individual’s ability to access social support for fear of negative responses. This is concerning given that women may prefer to seek abortion-related support from their partners, family members and friends. Hanschmidt et al. (2016) found that 91.7% women would prefer to seek support from their partners while 82.8% would turn to family and/or friends: figures much higher than the 43.8% to 47.9% who would seek professional help (e.g., counselling or mental health services). Stigmatizing reactions by these individuals may also exacerbate abortion-related distress. Overall, the impact of stigma on women’s post-abortion outcomes and pre-abortion decision-making seems two-pronged. Post-abortion, women may face “judgement, isolation…and community condemnation” while pre-abortion worries about these may “confound a woman’s decision to terminate a pregnancy” (Frohwirth et al., 2018, p.395).

### Abortion and Culture: Societal and Social Considerations

Compounding the issue of stigma is research suggesting that professional services are not exempt from stigmatizing service users. In a 2017 study by Altshuler et al., respondents reported “judgemental and dehumanizing” (p.112) interactions with doctors and feeling “susceptible to the negative judgements” (p.114) of other clinic staff and patients. This may deter women from discussing their concerns with their medical providers. This is concerning given Hanschmidt et al’s (2016) finding that women were likelier to approach their gynaecologist for abortion-related emotional problems than mental health services.

Another source of stigma is cultural. For example, in his description of Confucian philosophical perspectives on abortion (which may have an influence on Singapore’s Chinese majority), Ivanhoe (2010) contends that “an abortion…should be greeted with at least some degree of regret, as a manifestation of…the special joy and satisfaction of creating and raising children” (p.48). Women who experience little or no such emotional distress may hence be othered, impacting their psychosocial well-being in ways that the abortion, on its own, did not.

### The Present Research

In a review of the extant literature, it was noted that many of the studies on religion and abortion-related stigma, mental health and help-seeking were conducted in non-Asian contexts (e.g., North America, Europe). This raises questions regarding their generalizability to Asian nations such as Singapore where Christians are members of a minority religion that has not been historically culturally dominant. This suggests a research gap, given the influence of culture-specific beliefs, norms and values on social perceptions of abortion and an individual’s mental health and help-seeking (Gopalkrishnan, 2018).

These wider sociocultural differences may also have a trickle-down effect on the cultural and systemic practices of the local Church, possibly contributing to differences in individuals’ personal experiences and enactments of abortion stigma. Country-specific variations in the mental health care system may also result in differences in individuals’ help-seeking choices and options. This study sought to shed light on the concerns Christian women in Singapore may face, both in terms of their (anticipated) pre- and post-abortion emotional experiences and expressions, and their concerns and preferences in choosing support providers and practices.

To achieve these aims, the following four research questions were formulated:

1. How do Christian women in Singapore perceive abortion and the women who have them?
2. What help-seeking needs and preferences do Christian women anticipate having within the (hypothetical) context of an abortion for self?
3. What is the perceived willingness of others in the religious community to provide help pre- and post-abortion?
4. Is there a difference in the perceived importance of religious and non-religious (secular) support within the (hypothetical) context of an abortion for self?

## Method

### Design

Due to this study’s exploratory nature, a qualitative design was chosen to facilitate a broader and more in-depth exploration of the religion-related factors that influence an individual’s anticipated help-seeking needs, preferences and decision-making (Braun & Clarke, 2013). It also enabled discussions of perspectives falling outside religious norms.

To elicit participants’ anticipated psychosocial states and decision-making, this study used a hypothetical abortion scenario. There is both precedent and support in existing abortion research for the use of hypothetical designs. One such precedent is a study by Curlin et al. (2007) which posited that hypothetically based studies may yield valuable directions in which to take future research.

Other studies indicate that “personal experiences with abortion were not limited to women who had had the procedure themselves” (Herold et al., 2015 p.951) since women may “(think) about having an abortion at other times in their lives” (e.g., pregnancy scares). Smetana (1981) adds that “reasoning in the context of actual decisions about abortion is consistent with the more abstract and hypothetical reasoning about abortion observed from never-pregnant subjects” (p.222). Given this, a hypothetical design was employed on the consideration that perhaps “distinctions made between women who have had abortions and women who have not had abortions may not be useful” (Herold et al., 2015 p.951).

Inspired by Mitchell et al’s (2006) use of a cartoon vignette in a similarly hypothetical scenario-based abortion study, this study incorporated the use of a three-panel comic excerpt Hayes (2015). The comic depicted a woman facing the possibility of an unplanned pregnancy and contemplating between an abortion and pregnancy continuation. It provided participants with a gentle segue into the topics of unplanned pregnancy and abortion and a context in which to base their decision-making. The excerpt was devoid of abortion views and religious values.

### Participants

The final sample comprised eleven interviewees. All were female, ethnically Chinese, Singapore residents and baptized. A moderate sample size was used as it was considered “large enough to capture a range of perspectives…(without) drowning in data” (Braun & Clarke, 2013, p.45). Eight participants were recruited via snowball sampling; three were known others who volunteered participation. In addition to the interviewing techniques described above, given the sensitive nature of the topic, the use of snowball sampling further helped to encourage more open communication due to connections to mutually known others (Naderifar et al., 2017).

Purposive sampling was used to access a relevant sample with knowledge and experience of abortion perspectives within the Church (Braun & Clarke, 2013). The recruitment criteria were: self-identified as Protestant Christian; residing in Singapore at the time of the interview regardless of nationality and residency status; female; and between 21 and 49 years old (per the World Health Organization’s definition of adult female reproductive age). Participants were not required to belong to any particular denominations. All interested respondents who met the selection criteria were recruited. Due to the sampling methods used, the sample comprised demographically similar individuals. However, given the study’s small sample ***size*** and exploratory nature, this homogeneity proved useful as “homogenous sampling facilitates generating themes in small samples” (Braun & Clarke, 2013, p.50). A summary of participant’ details is given in Table 1.

**Table 1:** Participant background and demographic information

S. No	Age	Denomination	Church attendance (services)	Church participation (cell, bible study, volunteer groups)	Marital status	Children?
P1	33	Methodist	Weekly	Nil	Single	Nil
P2	30	Reformed	Weekly	Leadership	Married	1 child
P3	36	Pentecostal	Weekly	Regular	Married	1 child
P4	30	None (previously Pentecostal)	Nil	Nil	Single	Nil
P5	34	Independent	Weekly	Nil	Married	2 children
P6	30	Presbyterian	Monthly	Nil	Married	2 children
P7	28	Reformed	Weekly	Nil	Single	Nil
P8	29	Brethren	Weekly	Regular	Single	Nil
P9	33	Presbyterian	Weekly	Leadership	Married	1 child
P10	36	Methodist	Occasionally	Occasional	Married	1 child
P11	31	Anglican	Weekly	Occasional	Married	1 child

### Procedure

Semi-structured interviews, between one to two hours long, were conducted via Zoom (due to COVID concerns). A 26-item interview schedule and comic excerpt was used. This schedule was partially inspired by existing literature (Lifeway Research, 2015; Mitchell et al., 2006; Trybulski, 2006) and used as a guideline to ensure thematic similarity across interviews. Flexibility and discretion were used as necessary.

Due to ethical and privacy considerations, participants were not asked whether they had a history of abortion. Instead, a gentle pre-emptive warning was issued to inform participants of the possible psychological risk of participating, especially for individuals with abortion histories or difficult pregnancy-related experiences.

All participants provided informed consent. Prior to interview commencement, participants were reminded of their rights to cease participation at any time with no consequences and to withdraw unprocessed data and asked whether they were comfortable proceeding. Participants were also given an information sheet with the contact details of local abortion support services and informed to contact them in the event of post-interview distress. All interviews were recorded. For confidentiality, datasets were de-identified.

### Interviews and Analysis

Thematic analysis was chosen for its greater flexibility in examining thematic patterns across datasets while capturing thematic variations. It is posited that the researcher’s own experiences and perceptions influence qualitative data collection and analysis (Braun & Clarke, 2013). While data collection was carried out by the first author (interviewer), the interview schedule, collected data and analysis conducted during the period were reviewed regularly with the other authors who have different nationalities, religious affiliations and cultural backgrounds.

To manage and reduce the risk of unintended and undue interviewer influence on participant responses, interview techniques included pre-interview rapport-building, sensitive interviewing techniques and observation of and responsiveness to non-verbal cues. Care was taken to explore and discuss a range of views during interviews and the interviewer performed regular post-interview reflections. It is posited that being of the same gender and age range and having connections to a mutually known-other from the faith community helped with rapport-building and having more open conversations.

To maximize transcript familiarity and thematic accuracy and minimize the possibility of misinterpreting transcripts due to an absence of verbal cues, recordings were transcribed verbatim then read while listening to interview recordings. Initial codes were then generated and collated into emergent themes. Superordinate themes were identified based on coherent patterns in the codes and emergent themes and written up relative to the research questions.

## Results

A summary table of themes has been included in appendix (Table 2).

**Table 2:** Superordinate and emergent themes

Superordinate themes	Emergent themes
1. From “We are so precious to Him” to “A cell is not a life”: Attitudes towards “life” and abortion	1. Definitions of “life” affect one’s perception of abortion
	2. Nuances in the acceptability of abortion
	3. “God’s plan” and “What you really want”: Abortion-related advice-giving and personal decision-making
2. “Know what’s right and wrong”: Attitudes towards Christian women who have abortions	4. Holding the faith community to a higher standard
	5. Sins and sinners
	6. Reconciling differences (if any) in attitudes towards abortion and the women who have them
3. “How I feel about myself as a person”:	7. Psychological concerns
Anticipated abortion-related concerns	8. Faith concerns
	9. Social concerns
4. “A double-edged sword”: Common values and moral standards	10. Common values and moral standards prized in faith-based support
	11. Common values and moral standards as obstacles in seeking or accessing faith-based support
5. “Be slow to judge”: Church as a conditionally safe space	12. Behavioural and abortion-related conditions placed on persons who have abortions
	13. The culture of the church affects the safety of the church as a source of support
6. “I wouldn’t really want them to know”: Personal deterrents in seeking faith-based support	14. Faith is important but not necessarily the first priority
	15. Religious in-group membership as a double-edged sword
7. “I have to go back to the faith”: A preference for faith-based informal and formal support	16. Faith as theology
	17. Faith as in-group membership
8. “A secular service will be more neutral”: An openness to formal secular support	R. Independence in choice-making
	S. Logistical availability
9. “Give me practical steps”: The role of secular support in faith-based abortion recovery	T. Concerns in evaluating secular support
	U. Secular support as a complement to faith-based support

### Attitudes Towards “Life” and Abortion

This superordinate theme captures participants’ wide-ranging attitudes towards abortion. All eleven voiced that their attitudes were influenced by their definitions of “life”,

which were in turn influenced by their faith. P2 explained that “(Abortion) is not a right thing for a Christian to do (be)cause life is given by God”. For ten participants, these God-related perceptions of life extended into perceptions of abortion as “too much sin” (P10).

P1 clarified that the sin was one of “murder” due to the perception of life as predating conception and being tied to the faith-related concept of imbued divine meaning. P9 stated that “Even when your body is not fully formed, the Lord has already predestined your days and set you apart”.

For four participants, however, “life” was tied to the presence of a heartbeat and sentience. P11 noted that “A life is when you can start feeling things…let's say you are brain dead, are you still alive? Your heart is beating but your brain is not”.

For two participants, physical “life” was tied to foetal biological development though not necessarily to a heartbeat. P4 reflected that “Maybe it's because I look at plants grow. They don't have a heartbeat but there's something there that causes the seed to do its thing and grow roots and leaves”.

All participants voiced that their faith impacted their perceptions of the foetus. Nine perceived the foetus’ life as more important than the expectant person’s right to choose. In contrast, only two communicated that “Mother’s choice should come first” (P1). P11 cited the woman’s likely post-birth role as primary caregiver as justification for her right to choose.

“I think the main caregiver will be the mother…what’s the choice the fetus will want to make, that we will never know. But we know for that of the mother”. (P11)

Abortion was sometimes deemed justifiable. However, this was largely confined to maternal life-and-death situations and severe foetal deformities. Down syndrome, economic and social concerns and maternal psychological distress (e.g., depression, anxiety) were not considered justifiable grounds for abortion. When conception occurred under traumatic conditions (e.g., rape), abortion was uneasily accepted.

“It’s really those extreme cases where either I live or the child lives”. (P9)

“This child indeed has some very, very high chance of…(being) stillborn or have some sort of deformity in the heart or lungs”. (P5)

These nuances in the acceptability of abortion affected participants’ subsequent abortion-related advice-giving and personal decision-making. Although all participants indicated a consideration of individual circumstances, most (nine) indicated that they would advise pregnancy continuation in situations they viewed as not necessitating abortion.

### Attitudes Towards Christian Women Who Have Abortions

Participants tended to hold Christian women who have abortions to a higher standard than non-Christian women, a discrepancy attributed to a difference in values and religious knowledge. P6 explained that “If the person is not a believer then it’s hard to judge. And it's not (for) me to judge”.

Most participants also expressed the contingency of Christian women’s post-abortion acceptance upon the fulfilment of certain implicit demands. They are expected to repent and to feel “guilty” (P4), “grieved” (P5) and “the conflicts of her actions…that she has killed a baby” (P2). Conversely, women who feel positively about their abortions were characterized as “callous” (P4) and “not taking accountability” (P6).

Two participants, however, reported neutral perceptions of women who have abortions, citing their perception of God as offering empathic understanding and reproductive choice.

“Just tell God (that) I really can’t (continue the pregnancy)…I think God will be able to understand and empathize”. (P1)

“Maybe there's a certain decision that is more accepted by the Church but I also want to believe that He will let me make that decision myself”. (P11)

Three participants cited their professional experiences in their greater acceptance of women who undergo abortion. However, this acceptance did not necessarily imply abortion acceptance. One participant reported that “If she's (the client) in the dilemma, then I will talk to her more about pro-life… why don't you consider other options, do you have anything in mind, or yes, I agree that taking a life is such a horrible thing to do”.

### Anticipated Abortion-Related Concerns

All participants expected emotional difficulties pre- and post-abortion: Nervousness pre-procedure, a sense of loss post-procedure (i.e., of the foetus and a potential life change) and negative emotions stemming from their faith and unintended pregnancy.

“Pre, I think I’ll be very nervous. Post, I think there’ll be this sense of loss. Maybe I’ll feel very disappointed with myself…I don’t want this baby but…I feel so bad” (P1)

“Lots of guilt…Sadness. Regret...It’s against my conscience…against God’s law” (P2)

Participants also anticipated self-blaming cognitions and identity issues. P8 expected to see herself as “irresponsible (and having) committed a sin”. P4 expected a significant shift in her self-concept and to be “thinking about how I feel about myself as a person”. P9 noted a need to “make (the abortion) something meaningful or to help others” (e.g., by sharing her personal experiences with those considering abortion). Most participants anticipated feeling disconnected from God. Some also anticipated anger at God, while others anticipated a sense of shame and unworthiness. Three voiced concerns about how an abortion would affect their evangelism.

“It will be like a stumbling block to my non-believing friends”. (P2)

Overall, participants anticipated negative emotions and self-stigma, leading to perceived disruptions in their relationship with God. Most participants also reported a reluctance to seek out social support due to the anticipated social cost of abortion disclosures despite a simultaneous desire for support from God and their religious communities.

### Common Values and Moral Standards

Ten participants voiced a preference for social support from their faith communities, citing the importance of having common moral values with support providers and a perception of secular support as reflecting values unaligned with one’s own.

Nonetheless, participants anticipated that these common values could be a “double-edged sword” (P10) in accessing faith-based support, citing a fear of being negatively judged. Participants also reported a culture of silence within their churches regarding abortion, making it harder for them to identify safe sources of support and to comfortably seek it.

“Probably would have some judgmental views from people…for the premarital (sex)...the abortion is a killing of life which is kind of like a worser thing…Already wrong and now you’re doing something worse…It’s like (a) double blow” (P2)

Overall, the same quality (common values and morals) that made faith-based support appealing also reduced its accessibility. This contributed to participants’ anticipation of conditional help-seeking (reflected in the next theme).

### Church as a Conditionally Safe Space

This theme reflects participants’ perceptions of their religious communities as either conditionally safe or completely unsafe for help-seeking. Alongside the difficult emotions (e.g., grief and guilt) that one is expected to feel about their abortion, individuals seeking support are expected “to repent” (P2) and be penitent before receiving social or emotional support from their faith communities. Stipulations on the type of support one could expect were also voiced.

“Participating is sinning…Emotional and spiritual (support such as prayer), yes…Practical help …it's very hard (to obtain)”. (P6)

Participants’ willingness to seek church-based support was similarly conditional, hinged upon the perceived social acceptability of the abortion within their religious communities.

“I might not be able to continue going to church”. (P8)

“I don't think that it will be so safe…Even if (some people) are pro-choice…as a church, I don't know whether (pro-life) is something they have to portray”. (P11)

Overall, most participants anticipated social challenges and stigma and expressed doubts about their religious communities’ effectiveness as safe sources of support. These (and other) deterrents are examined in greater detail in the next theme.

### Personal Deterrents in Seeking Faith-Based Support

In choosing support sources, participant voiced the following criteria: Trustworthiness, open acceptance and close personal ties. Possible sources listed included Christian family members and close friends who would be “more than willing to support…(and) truly understand the scenario” (P2) and who would also “not make me feel worse than I already made myself feel” (P9). Participants anticipated being very careful with their selection process, citing a fear of gossip, judgement and non-acceptance. Some participants further required that the supportive person be “not from the same church” (P1). P4 explained that “(If) you're not going to the church I'm going to, (you) can't gossip because nobody will know me”. Participants also anticipated a degree of undue influence by others.

“If I wasn't a Christian…that decision of whether or not to undergo an abortion might have been easier…but because I am, it's no longer just my own decision…I also had to consider what would my church think, what would my church leaders think, what would my pastor say…I wouldn’t want them to tell me what to do but what I choose to do at the end will also be having taken into consideration how you impact them”. (P11)

Overall, most participants preferred to seek informal support from their wider faith communities outside of their respective churches, citing a blend of common values and privacy. Four participants also reported an openness to non-religious informal support in order to “have an open conversation” (P1) with greater perceived neutrality.

### A Preference for Faith-Based Support

Despite challenges, participants reported a strong preference for faith-based support. Theologically, faith-based support was perceived as addressing the issues underlying abortion-related psychological (e.g., self-stigma and painful emotions) and spiritual (e.g., a broken relationship with God) difficulties. Participants described it as offering a sense of salvation and (divine) forgiveness, the remission of sins and, consequently, greater impact in repairing the damage inflicted by stigma on one’s sense of self and identity.

“The assurance from God’s word is…eternal. It’s not just based on how I feel, how I think…It is really what God thinks…It’s more lasting and more reassuring…Knowing that my guilt and shame is committed to God and I’ve been forgiven by God” (P2)

Socially, faith-based support provided a sense of being implicitly understood and related to. It also served as a sign that the individual still retained social acceptance and membership within her religious community.

“It’s their identity…They are Christian…Because we belong there, you feel like your people, your own people reassure you...you will feel very comforting”. (P1)

Three participants, however, opted for faith-based formal services over informal support, citing their prioritized need to ensure privacy and confidentiality.

“Privacy matters…If I go to church, I’ll see (other churchgoers) weekly…I don’t feel comfortable…VWO, you’re getting their service…Once service done, it’s done”. (P1)

### An Openness to Formal Secular Support

Despite a penchant for faith-based support, most participants were open to formal secular support. Reasons cited included wanting a temporary separation from God and the Church to avoid emotional pain and for greater independence and privacy in decision-making.

“(Faith-based support) would make me feel guilty…The last thing I want to hear is…we will pray for you”. (P11)

“I'll be concerned that they have their own views and they press it onto me... I wouldn't want to be forced to not have an abortion”. (P4)

Participants also voiced an openness to formal support groups (faith-based and secular) as additional supportive spaces citing anticipated social and logistical difficulties in accessing faith-based abortion-related support and its limited availability.

“Depending on whether there might be support in the faith-based community…(e.g.,) a psychiatrist or doctor who is also a Christian…Depends on the options I have”. (P7)

Nonetheless, participants’ willingness to use formal secular services came with caveats.

### The Role of Formal Secular Support in Faith-Based Abortion Recovery

Participants emphasized that the role of formal secular support was to be a complement for faith-based support and that any non-religious formal support provided was to be completely neutral. Mindfulness-based therapies were singled out as of particular concern.

“Even secular (support), they might have their own religions as part of that. I don’t want myself to be imposed by things that I’m not assured of…Like mindfulness”. (P3)

Participants also expressed a hopefulness that formal secular services might be better trained to “help and understand the needs” (P9) of women who have had abortions.

“(Secular support) has to...give me practical steps”. (P5)

“It's more the quality of the counsellor... Because if their skills are terrible and they just quote Bible verses at me then (laughs)”. (P4)

Overall, participants expressed a degree of wariness towards the content of secular support. Quality of the support service was also given a significantly higher weightage by participants in their consideration of formal secular support than in formal faith-based support.

## Discussion

In line with the summary of Singapore-specific findings in the World Values Survey (Mathews et al., 2021), most participants communicated unfavourable views of abortion and the women who have them. Additionally, similar to findings by Frohwirth et al. (2018), all participants anticipated experiences of felt, enacted and internalized stigma. While it should be acknowledged that “harsher self-judgements” (Cockril & Nack, 2013, p.980) and feelings of unworthiness are not necessarily maladaptive in Christian religious contexts (e.g., given Christian teachings repentance for the remission of sins), participants’ anticipation of a continuous broken relationship with God and a sense of spiritual exclusion indicate self-stigmatization as per the definition provided by Cockrill and Nack (2013). Such sustained self-stigmatization may contribute to longer-term mental health problems.

Participants also expected to be judged by other Christian women more harshly for elective abortion. Findings suggest that support-giving and acceptance may be hinged upon the fulfilment of implicit behavioural (e.g., repenting) and circumstantial (e.g., biomedically necessary) conditions. This impacted participants’ perception of faith-based support’s safety and accessibility, manifesting in their post-abortion decisions and secrecy. This aligns with existing research findings (Frohwirth et al., 2018) that Protestant women largely anticipated unfavourable responses by their religious communities and were likelier to maintain silence on past abortions. Participants made exceptions only for biomedically necessary abortions, which were unlikely to be accompanied by a sense of shame and fear of social reprisals.

Overall, despite a preference for faith-based informal support, participants opted for formal faith-based and secular services, albeit with caveats. The use of formal secular services was anticipated to be likelier during post-abortion avoidance of God and/or the Church. Nonetheless, there remains a risk that participants’ expectations of formal secular services (e.g., of neutrality or objectivity) may be frustrated (Altshuler et al, 2017). Additionally, participants who expressed perceptions of God as offering reproductive choice and having a more empathic attitude towards abortion also tended to anticipate higher decision satisfaction and better coping in the long term. This may align with research suggesting that “social messages and support groups that encourage women to cognitively reappraise an abortion in a more positive or benign way may lead to improved emotional responses” (Major et al., 2009, p.868).

### Implications

The results obtained suggest a number of therapeutic considerations when working with Protestant Christian women with past abortion experiences. The first reflects a clinician–advocate approach and considers addressing social risk factors in Christian and non-Christian women’s post-abortion outcomes (e.g., through a trickle-down effect to the faith community of outreach within the wider public). Apart from addressing stigmatizing stereotypes, outreach efforts would ideally aim to address concerns pertaining to physical (e.g., concerns that abortion harms fertility) and mental health (e.g., debilitating self-stigma), and help to normalize wider public discourse on abortion-related needs and concerns.

The second consideration reflects a clinician–practitioner approach and concerns intrapersonal psychological risk factors and outcomes. Although not voiced by any of the participants in this study, it is possible that Christian women experiencing distressing levels of abortion-related self-stigmatization may find the reframing of the “sin” of abortion potentially helpful (e.g., in terms of the degree of agency she had, the extent to which she truly had a choice given her circumstances, and/or in terms of the possibility of redemption in spite of inevitable and original sin). Although some participants stipulated the complete religious neutrality of formal services as a prerequisite for using them, all participants voiced a desire to eventually reestablish a connection to their faith and faith communities. Where appropriate, this focus on spirituality can also be integrated therapeutically (e.g., adopting a greater focus on unchanging divine forgiveness as a counterpoint to persistent self-stigmatization).

The third consideration reflects a service linkage approach and concerns the provision of additional support measures to further meet Christian women’s abortion-related psychosocial needs. Given that an abortion may risk the exclusion of some churches as safe spaces for support-seeking, additional new spaces may be helpful. These may be accessible through the use of faith-based and secular formal services (e.g., psychotherapy and support groups). These grant women access to social and psychological support while providing privacy and protection against the social consequences of abortion disclosure in their everyday lives.

Additionally, churches wishing to offer safe spaces without deviating from their position on abortion may consider training programmes for faith leaders, empowering them to provide supportive counselling, build destigmatized spaces for conversations and support-seeking, and provide referrals to secular healthcare providers (Molina, 2021).

To provide further support for pregnancy continuation, churches may also consider providing non-judgmental pregnancy-related support for individuals (women and men) facing unplanned pregnancies. Such measures may include consensual prayer, religiously informed pregnancy and parenting programmes and mentorships and some degree of financial support where possible. The discussion of abortion from the pulpit with “love and compassionate support” (Holloway, 2022) may also help to address a “fear of judgement and rejection” and the normalization of abortion-related conversations (e.g., abortion-related needs, dilemmas and difficulties).

### Limitations

Although care was taken in the design and implementation of this study, the following limitations were noted. Firstly, due to time constraints and difficulty finding participants, the sample ultimately consisted of eleven interviewees. While this fulfilled Braun and Clarke’s (2013) recommendation (i.e., at least ten interviews), a larger sample might have yielded greater thematic diversity and repetition.

Secondly, the sample consisted of racially Chinese females of a similar age range and who were largely Singaporean. Hence, the sample cannot be considered representative of Singapore’s Christian community. Christians of other races, nationalities and ages may hold dissimilar views. Participants may also have been among a subset of Christian women with more socially acceptable views. Those with less conventional views may be less forthcoming.

A third limitation may lie in the lack of a second coder due to time constraints. As mitigation, all results, generated themes and (coded and uncoded) transcripts were checked and verified by all authors of this study before they were accepted and written up.

### Future Research

Demographically, the sample did not include individuals from different age groups or with non-heteronormative or non-cisnormative orientations. Christians of other generations may present with variations in abortion views, and the LGBTQIA + community in particular remains underrepresented in abortion and reproductive research.

Secondly, three participants in this study provided insights into how their abortion views affected their professional interactions with individuals contemplating abortion. Further research may help in understanding how Christian abortion support professionals reconcile their beliefs with their job requirements and the impact of their work on their clients.

As mentioned by a participant (P11), “Even if (some people) are pro-choice…as a church, I don't know whether (pro-life) is something they have to portray”. Although not explored in this study, such dissonance between private beliefs and public stances may be experienced by both laypersons and church staff (e.g., clergy, pastoral staff). As such, a third possible area of future research may lie in exploring the abortion views of church staff in Singapore, the extent of such dissonance (if any) and the factors (e.g., personal, occupational) contributing to it.

## Conclusion

This study sought to explore the impact of religion on Christian women’s abortion-related help-seeking preferences and concerns as well as the perceived safety of the faith community as a space for support-seeking. Findings revealed that although all participants prioritized faith-based support, many anticipated that an abortion would make it challenging for them to seek such support both pre- and post-procedure. To enhance the perception of the Church as a safe space for abortion-related support-seeking, various measures can be employed to provide support to individuals (women and men) facing unplanned pregnancies or who have already had abortions and to normalize abortion-related discussions. Overall, a cooperative (rather than exclusionary) approach between faith-based and secular organizations may be more helpful within the Singapore context.

## Interview Schedule

1. What is your opinion of abortion? Why?
2. How do you perceive women and girls who have abortions? Why?
3. Does your church perceive abortion and those who have them in a similar way? If it differs, how does it differ?
4. In your opinion, are there any situations in which abortion might be acceptable?
5. If you were to meet the comic’s main character, how would you advise her? Why?
6. If your friend or sister had an unintended pregnancy, how would you advise her? Why?
7. If you were to have an unintended pregnancy, what would you do? Why?
8. If you were to have an abortion in that situation, how do you think you would feel?
9. Do you think that your own religious views would influence how you feel and think about the abortion? If so, in what ways?
10. Do you think that the religious views of others would influence how you feel and think about the abortion? If so, in what ways? Would they try to impose their views on you?
11. If you were to have an abortion, who would you discuss it beforehand with or disclose it afterwards to anyone? Why?
12. Comparing both settings (within and outside of your faith community), where would you feel more supported about the abortion? In what way?
13. How likely do you think people in your church are to provide help pre- and post-abortion?
  - Practical help (e.g., financial help, giving a lift to and from abortion-related clinic and medical appointments)
  - Emotional and social support (e.g., providing companionship in and out of Church through difficult abortion-related feelings)
  - Spiritual support – this can be linked with emotional and social support (e.g., informal prayer requests to close Church friends and pastors)
14. Between faith-based and secular sources of reassurance (e.g., for feelings of shame and guilt), which do you think would be more impactful? Why?
15. If both the faith and secular spaces were equally non-judgmental and supportive, where would you prefer to seek support from? Why?
16. Some people hold the view that the rights of the mother (e.g., to choose whether or not to abort or continue the pregnancy) should take precedence over the fetus. What do you think? Why?
17. Do you think life begins at conception, at birth or sometime in between? Why?
18. How often do you attend services? Referring to our earlier conversation, would you expect this to change after the hypothetical abortion? If so, why?
19. Outside of services, do you participate in any church activities? Referring to our earlier conversation, would you expect this to change after the hypothetical abortion? If so, why?
20. What is your denomination?
21. Are you a baptised member of any church?
22. Do you currently have any children? If yes, how many?
23. What is your age?
24. What is your race?
25. What is your nationality?
26. Where do you currently reside?

## Footnotes

1 Despite the use of the term “Termination of Pregnancy” in the act’s name, it is rarely used colloquially in Singapore to refer to elective induced abortion. The term “abortion” is more commonly used in everyday speech instead, often without the specifiers “elective” and “induced”. To reflect this, the term “abortion” in this study refers to elective induced abortion.
